# Holey Graphene: Topological Control of Electronic Properties and Electric Conductivity

**DOI:** 10.3390/nano11051074

**Published:** 2021-04-22

**Authors:** Pavel V. Barkov, Olga E. Glukhova

**Affiliations:** 1 Institute of Physics, Saratov State University, 410012 Saratov, Russia; barkovssu@mail.ru; 2 Institute for Bionic Technologies and Engineering, I.M. Sechenov First Moscow State Medical University (Sechenov University), 119991 Moscow, Russia

**Keywords:** electrical conductivity, electrical conductivity anisotropy, holey graphene, graphene, computer simulation

## Abstract

This paper studies holey graphene with various neck widths (the smallest distance between two neighbor holes). For the considered structures, the energy gap, the Fermi level, the density of electronic states, and the distribution of the local density of electronic states (LDOS) were found. The electroconductive properties of holey graphene with round holes were calculated depending on the neck width. It was found that, depending on the neck width, holey graphene demonstrated a semiconductor type of conductivity with an energy gap varying in the range of 0.01–0.37 eV. It was also shown that by changing the neck width, it is possible to control the electrical conductivity of holey graphene. The anisotropy of holey graphene electrical conductivity was observed depending on the direction of the current transfer.

## 1. Introduction

A new unique structural derivative of graphene, “holey graphene” (HG), also called “graphene nanomesh” or “hole-matrixed graphene”, is finding more and more applications [1,2,3,4,5,6,7]. The unique physical and chemical properties of HG are provided by the edge atoms near nanoholes. This area can be easily functionalized by different chemical groups that expand HG’s range of applications since functionalized graphene materials are more promising for new polymer composite design, and for biological applications. The presence of holes in graphene changes the properties of this 2D material and opens wide the prospects for application in nanoelectronics. HG is an easy-to-manufacture nanostructure with a band gap that can be tuned. HG-based field-effect transistors can support currents almost 100 times greater than individual devices based on graphene nanoribbons [8]. HG is also promising as a basic element for supercapacitors and batteries since its reticulation allows lithium ions to freely penetrate into the electrode [9,10,11]. Of course, the mesh structure determines this material as high-performance membranes that can be used for gas separation [12] and water purifiers [13,14,15].

The main parameters of HG are periodicity (the distance between the centers of neighbor holes) and neck width (the smallest distance between neighbor holes).

There are HG with round, triangular, and rectangular holes [16,17,18,19,20]. Most often, in a real experiment, HG with round holes are synthesized. The DFT study of energy gap dependency on the neck width showed that HG with round holes had the bigger energy gap compared to HG with triangular and rectangular holes [16]. Winter et al. found that HG with round holes had high mechanical stability and could cover intact areas up to 2500 mm^2^ [21]. It was revealed that HG with round holes had isotropic elastic properties in contrast to HG with elliptical holes [22]. Another advantage of HG with round holes is the possibility to grow nanotubes of different chirality on its surface [23,24]. Earlier, authors of this study showed that that the growth of single-walled carbon nanotubes (6,6) and (9,9) in HG round-shaped holes with a size of approximately 0.8–1.2 nm was energetically favorable [25,26].

The aim of this work was to study the dependency of the electrical conductivity in the HG with round holes on the neck width. In the zigzag direction, the neck width was increased from 0.74 nm to 5.18 nm, while in the armchair direction the neck width was increased from 0.99 nm to 5.25 nm.

## 2. Materials and Methods

**Figure 1:**
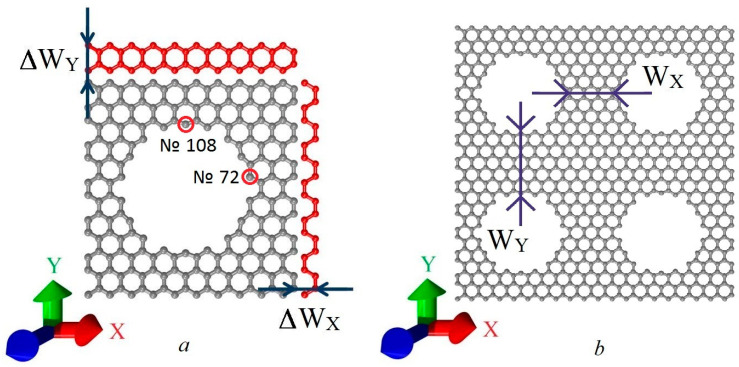
(**a**) HG supercell with the minimum possible steps for increasing the neck width along the zigzag (ΔW_X_) and the armchair (ΔW_Y_) direction; (**b**) the neck width along the zigzag (W_X_) and the armchair (W_Y_) directions.

In this paper, we considered an atomistic model of HG with round holes of 1.2 nm in diameter. Figure 1a shows its supercell and the minimum possible steps for increasing the neck width: ΔW_X_ = 0.24 nm for the zigzag direction and ΔW_Y_ = 0.42 nm for the armchair direction. The length of the supercell in the X direction (zigzag edge) was 2.46 nm and in the Y direction (armchair edge) 2.55 nm. These are the smallest dimensions at which the supercell is energetically stable. Atoms № 72 and № 108 were the edge atoms near the hole. Figure 1b shows the initial minimum neck widths along the zigzag (W_X_) and along the armchair directions (W_Y_). The study was performed in two stages. At the first stage, the neck width W_X_ was changed from 0.74 nm to 5.18 nm with the minimum possible step ΔW_X_, while the neck width W_Y_ remained unchanged and equaled 0.99 nm. At the second stage, the neck width W_Y_ was changed from 0.99 to 5.25 nm with the minimum possible step ΔW_Y_, while the neck width W_X_ remained constant and equaled 0.74 nm.

The function of electrical conductivity, G, was calculated by the Green–Keldysh nonequilibrium function method [27] with application of the Landauer–Butticker formalism, which allowed us to study the quantum transport of electrons while taking into account the elastic scattering of electrons in inhomogeneities [28]. The electrical conductivity is written as

(1) $$G=2e^{2}/h\int_{-\infty }^{\infty }T\left(E\right)F_{T}\left(E-E_{F}\right)\mathrm{dE},$$

where T(E) is the electron transmission function, E_F_ is the Fermi energy of contacts connected to the considered object, e is the electron charge, h is Planck’s constant, e^2^⁄h is the quantum of conductivity (the value of conductivity for a single channel), and F_T_ is the function that determines the value of the temperature broadening. The multiplier “2” takes into account the spin. The electron transmission function is defined as follows:

(2) $$T\left(E\right)=\mathrm{Tr}\left({Г}_{S}\left(E\right)G_{C}^{A}\left(E\right){Г}_{D}\left(E\right)G_{C}^{R}\left(E\right)\right),$$

where $G_{C}^{A}\left(E\right)$ and $G_{C}^{R}\left(E\right)$ are the advanced and the retarded Green matrices describing the contact with the electrodes, respectively; ${Г}_{S}\left(E\right)$ and ${Г}_{D}\left(E\right)$ are the level broadening matrices for the source and drain, respectively. The transmission function, T(E), and the electrical conductivity, G, were calculated by the Kvazar–Mizar 1.0 software package (Saratov State University, Saratov, Russia) [29,30].

The energy, E, of the multi-electron system was determined by the self-consistent charge density functional tight-binding (SCC DFTB) method [31]. The SCC DFTB method solves a system of one-electron Kohn–Sham equations [32] written in the following form:

(3) $$H_{\mathrm{KS}}\Psi_{i}=\varepsilon_{i}\Psi_{i},$$

where Ψ_i_ is the wave function of i-th electron, ε_i_ is the energy of i-th electron, and H_KS_ is the Hamiltonian.

At the stage of the system’s total energy calculation, the tight binding approximation, which is included into the DFT model using perturbation theory, is applied. Within the SCC DFTB method, the expression for the total energy of the system is written as follows:

(4) $$E_{\mathrm{TOT}}=E_{\mathrm{OCC}}+E_{\mathrm{SCC}}+E_{\mathrm{REP}},$$

where E_OCC_ is the energy of occupied electron states, E_SCC_ is the energy of the electron interactions, and E_REP_ is the energy of interaction between particle pairs due to repulsive forces.

The SCC DFTB method takes into account the effect of electron density fluctuations on the total energy of the system. The distribution of atomic charges is determined from the population analysis according to the Mulliken scheme [33,34,35]. Accounting for the self-consistent charge distribution makes it possible to significantly improve the accuracy of calculations for polyatomic systems containing covalent and ionic bonds. The choice of the SCC DFTB method for calculating the total energy of a multi-electron system was due to the polyatomic nature of the considered supercells containing 1000 or more atoms. As is known, it is very resource-intensive to study polyatomic cells using DFT, so the SCC DFTB method is preferred. The system’s total energy E was calculated by the DFTB+ 20.2 software package (University of Bremen, Bremen, Germany) [36,37].

The search for the equilibrium configuration of the considered supercells was performed in the form of a double optimization, which involved minimizing the total energy of the system over the lengths of the translation vectors L_X_, L_Y_, and over all coordinates of the supercell atoms within the SCC-DFTB method. All calculations were performed at the temperature of 300 K.

## 3. Results

**Figure 2:**
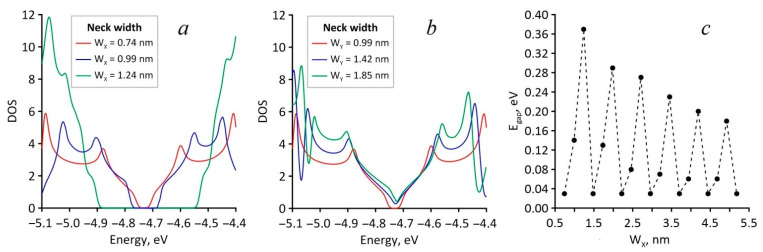
Electronic properties of HG: (**a**) DOS with increasing of W_X_ (zigzag direction); (**b**) DOS with increasing of W_Y_ (armchair direction); (**c**) dependence of energy gap on W_X_.

Initially, for different values of the HG necks, we calculated electron characteristics such as the energy gap (E_gap_), the Fermi level (E_F_), and the density of electronic states (DOS). Figure 2a,b shows the HG DOS graphs for three different values of the neck width with its gradual increase in the zigzag and armchair directions, respectively. With a gradual increase in the neck width in the zigzag direction (W_X_), the width of the energy gap experienced a jump-like change in the range from 0.03 to 0.37 eV (Figure 2a). At the same time, for the values W_X_ = 0.74 × N nm (N = 1 ÷ 7), the energy gap remained unchanged and equal to 0.03 eV. The width of the energy gap reached the highest value at the neck width of 1.24 nm. For all cases of gradual increases in the neck width along the armchair direction (W_Y_), the width of the energy gap varied within 0.02 ± 0.01 eV, as shown in Figure 2b. For both directions of increase in the neck width, the Fermi level varied within −4.71 ± 0.02 eV. Thus, on the basis of the DOS analysis, it was concluded that HG demonstrated anisotropy of electronic properties.

**Figure 3:**
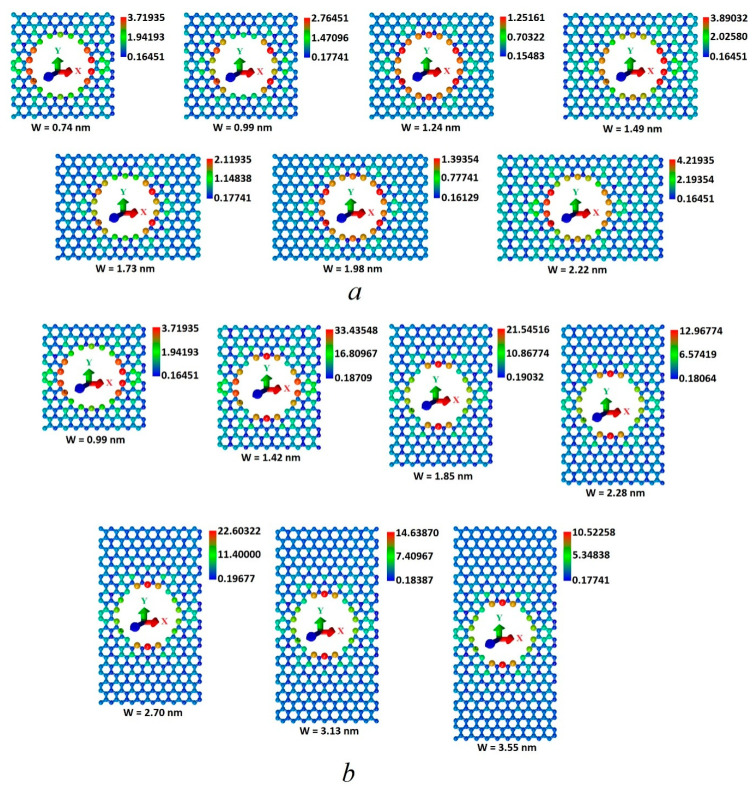
Maps of the HG’s LDOS distribution in the case of the neck width increasing along the zigzag (**a**) and armchair (**b**) directions (in LDOS units of a pure graphene atom).

**Figure 4:**
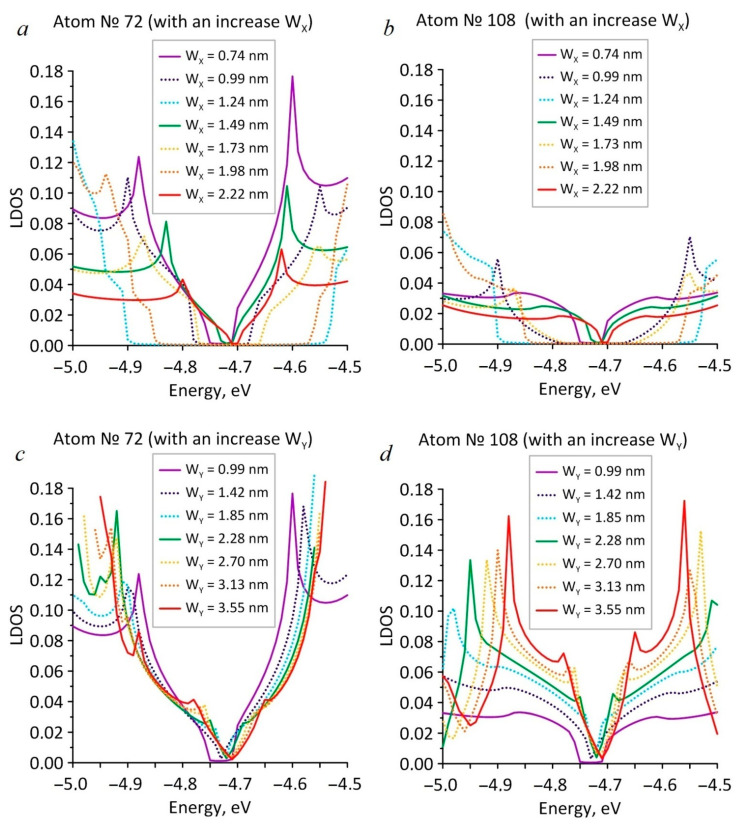
LDOS plots for atoms № 72: (**a**) with the increasing of W_X_; (**b**) with the increasing of W_Y_; and № 108: (**c**) with the increasing of W_X_; (**d**) with the increasing of W_Y_.

In order to understand the nature of the anisotropic appearance, the distribution of the local density of states (LDOS) over the supercell atoms and the LDOS distribution in the region of energies close to the Fermi level were calculated. We assumed that the edge atoms in the hole would have increased LDOS values, having a significant effect on the LDOS distribution of all the supercell atoms and, consequently, on the electronic properties of HG. Figure 3 shows the maps of the LDOS distribution, calculated at the Fermi level, for different values of the neck width with its gradual increase along the zigzag (Figure 3a) and armchair (Figure 3b) directions. The color palette displays the LDOS values in LDOS units of a pure graphene atom (3.1*10^−4^ absolute units at the Fermi level). As expected, the atoms at the edges of the hole had a maximum LDOS value (red). Atoms with LDOS values in the intervals of 0.7–2 relative units (Figure 3a) and 2–17 relative units (Figure 3b) formed green paths between neighboring HG holes along the armchair direction (1 relative unit = 3.1*10^−4^ absolute units at the Fermi level for pure graphene). Along the zigzag direction, both in the cases of the W_X_ and W_Y_, growth atoms with LDOS values of less than 0.2 relative units (blue) were predominantly located. Such distribution of the LDOS values across the atoms in both directions is the reason for the anisotropy of the HG electronic properties. The LDOS values on the atoms at the edges of the hole and on the atoms of the green track in the case of increasing the neck width along the zigzag direction were several times less than the LDOS values on similar atoms during increasing the neck width along the armchair direction. Figure 4 shows the LDOS graphs for two separate atoms, № 72 and № 108, located at the edges of the HG hole. Atom № 72 was located at the edge of the armchair type, and atom № 108 was located at the edge of the zigzag type. In Figure 4a,b it is seen that in the case of the increase in the neck width along the zigzag direction, the LDOS profiles for both atoms repeated the DOS profiles of HG near the Fermi level, demonstrating a jump-like change in the width of the energy gap. Similarly, Figure 4c,d shows that in the case of the increase in the neck width along the armchair direction, the LDOS distributions for atoms № 72 and № 108 were similar to the DOS distributions with an increase in the W_Y_, and the width of the energy gap changed monotonically. However, for both cases of W_Y_ and W_X_ increasing, the LDOS profiles for atoms № 72 were two to three times larger than for atom № 108.

**Figure 5:**
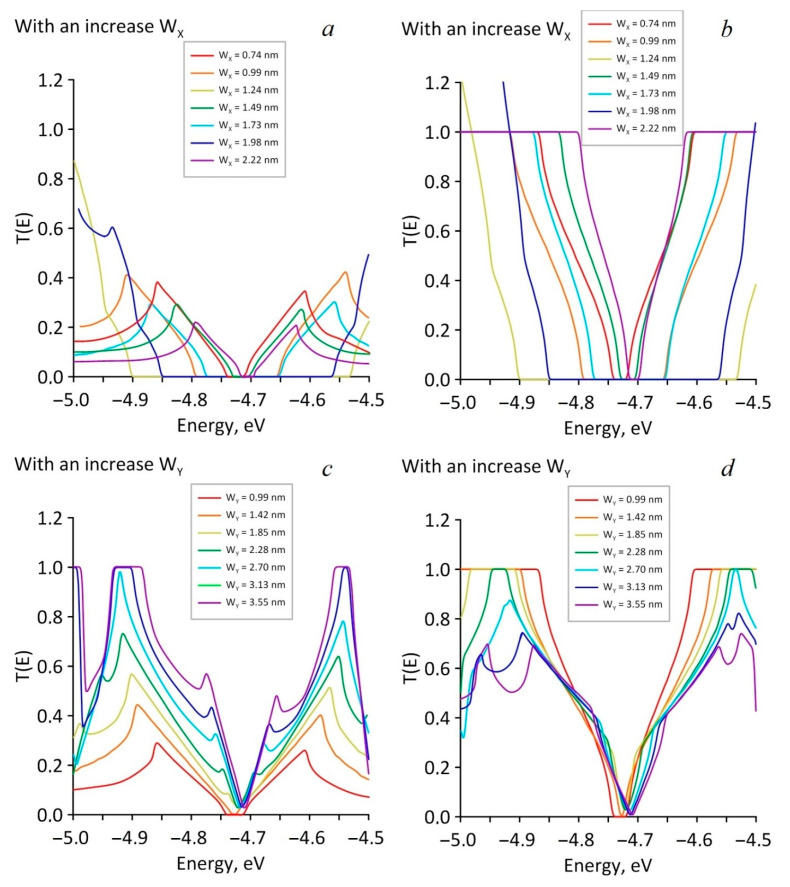
Graphs of HG transmission function T(E) (in e^2^/2h) in the case of Wx increasing: (**a**) for the current transfer along the zigzag direction; (**b**) along the armchair directions; and in the case of Wy increasing: (**c**) along the zigzag direction; (**d**) along the armchair directions.

Next, the transmission functions T(E) were calculated. These were necessary to determine the electrical conductivity (G). The profile of transmission functions was determined by the profile of the DOS plot. It was logical to assume that the presence of the energy gap in the DOS profile would lead to the appearance of a zero interval in the transmission function, and that the established pattern of change in the energy gap width with an increase in the neck width would also appear for the transmission function T(E). The obtained results are shown in Figure 5. Figure 5a,b shows graphs of the transmission function with the increasing of W_X_ in the case of current transfer along the zigzag (a) and armchair directions (b). Figure 5c,d shows the T(E) graph with an increasing of W_Y_. It follows from Figure 5 that in the armchair direction (Figure 5b,d), the electrical conductivity of HG near the Fermi level was higher than in the zigzag direction (Figure 5a,c), since in the armchair direction the transmission function T(E) reached 1, and in the zigzag direction its value didnot exceed 0.6. It can be assumed that the reason for the difference in the behavior of the T(E) function is the localization of electrons, which leads to a significant increase in the local density of electronic states at the edges of the holes and, as a result, to a redistribution of the electron density over all the atoms of the structure.

**Figure 6:**
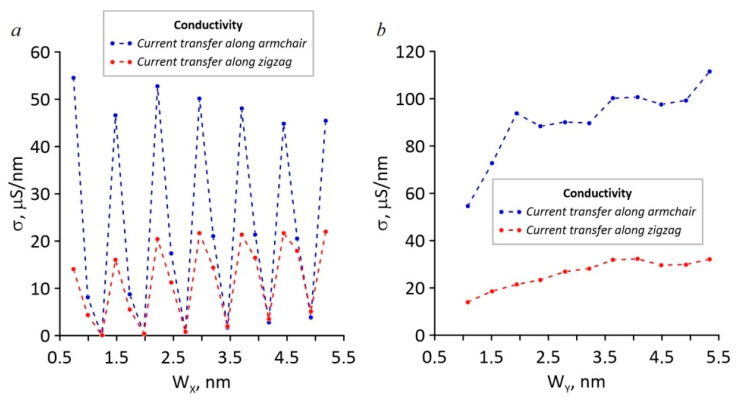
The dependency of the specific conductivity of HG on the neck width during an increase along the zigzag (**a**) and the armchair directions (**b**).

The values of the specific electrical conductivity calculated on the basis of T(E) are shown in Figure 6. The analysis of the presented plots showed that during an increase in the neck width along the zigzag direction, there was a jump-like change in the specific conductivity in the case of current transfer both along the zigzag and armchair directions (Figure 6a). At the same time, every third value of the specific electrical conductivity for current transfer along the armchair direction was almost three to four times greater than every third value of the specific electrical conductivity for current transfer along the zigzag direction. The greatest specific electrical conductivity of HG was reached in the case of current transfer along the armchair direction during an increase in the neck width along the armchair direction (Figure 6b). In the case of current transfer along the zigzag direction, the specific conductivity of HG was several times less.

## 4. Conclusions

In the result of mathematical modeling, new knowledge was obtained about the effect of the width neck on the electronic properties of holey graphene with round holes of approximately 1.2 nm in diameter. On the basis of the DOS distributions analysis, it was concluded that, depending on the neck width, HG demonstrated a semiconductor type of conductivity with an energy gap varying in the range of 0.01–0.37 eV.

It was also shown that by changing the neck width, it is possible to control the values of the HG electrical conductivity. It was discovered that the anisotropy of the electrical conductivity depends on the direction of current transfer. The highest specific electrical conductivity of HG was reached in the case of current transfer along the armchair direction during an increase in the neck width along the armchair direction.

Thus, according to the simulation results, it is predicted that holey graphene can become the basis for the element base of semiconductor devices.
